# Nasal Nitric Oxide Levels in HIV Infection: A Cross-Sectional Study

**DOI:** 10.1155/2018/7645125

**Published:** 2018-01-09

**Authors:** Cecilia T. Costiniuk, Vikram Mehraj, Jean-Pierre Routy, Christina de Castro, Natale Wasef, Mohammad-Ali Jenabian, Syim Salahuddin, Bertrand Lebouché, Joseph Cox, Jason Szabo, Marina Klein, Larry Lands, Adam J. Shapiro

**Affiliations:** ^1^Chronic Viral Illnesses Service and Research Institute, McGill University Health Centre (MUHC), Montreal, QC, Canada; ^2^Department of Medicine, National University of Ireland, Galway, Ireland; ^3^Department of Biological Sciences and BioMed Research Centre, Université du Québec à Montréal (UQAM), Montreal, QC, Canada; ^4^Division of Pediatric Respiratory Medicine, McGill University Health Center Research Institute, Montreal, QC, Canada

## Abstract

**Introduction:**

Low levels of nasal NO have been associated with increased propensity to rhinosinusitis and respiratory tract infections. Our objective was to describe nasal NO levels in HIV-infected individuals versus healthy controls and determine possible risk factors for reduced nasal NO levels.

**Materials and Methods:**

HIV-infected individuals and healthy controls were recruited. Participants underwent nasal NO testing by standardized methods using a CLD88 chemiluminescence analyzer and completed the Sinonasal Outcome Test-20 (SNOT-20) on symptoms of rhinosinusitis.

**Results:**

Participants included 41 HIV-infected individuals with suppressed VL on antiretroviral therapy (ART group), 5 HIV-infected individuals with detectable VL off ART (viremic group), and 12 healthy controls (HC group). Mean nasal NO level was 253 (±77) nL/min in the ART group, 213 (±48) nL/min in the viremic group, and 289 (±68) nL/min in the HC group (*p* = 0.133; ANOVA). There was no correlation between nasal NO level and VL in viremic individuals (*r* = −0.200; *p* = 0.747). Differences were observed in mean total points on the SNOT-20 which were 19 (±16)/100, 18 (±26)/100, and 4 (±4)/100 in the ART, viremic, and HC groups, respectively (*p* = 0.013; ANOVA).

**Conclusion:**

Healthy individuals, HIV patients on ART, and viremic individuals off ART display similar nasal NO levels. However, rhinosinusitis symptoms remain prominent despite ART-treatment.

## 1. Background

Although antiretroviral therapy (ART) reduces one's risk for various infections, rhinosinusitis remains one of the most common complications of HIV infection and is experienced by up to 68% of patients during their infection [1]. Induced by infectious stimuli, nasal nitric oxide (NO) is an important innate defense against various respiratory pathogens, and low NO levels have been associated with increased propensity to rhinosinusitis and other respiratory tract infections [2]. In a single study to explore nasal NO levels in HIV-infected individuals, performed by Palm and colleagues during the early ART era, individuals with HIV had significantly reduced levels of nasal NO [3]. Since the study by Palm and colleagues during the early ART era, no subsequent studies have examined nasal NO levels in individuals receiving long-term ART. Our objective was to describe nasal NO levels in HIV-infected individuals on modern, effective ART relative to levels observed in HIV-infected individuals not on ART and healthy controls. A secondary objective was to determine possible patient characteristics for reduced nasal NO levels in HIV-infected individuals.

## 2. Materials and Methods

Following the ethical approval by the McGill University Health Centre (MUHC) Research Ethics Board, 3 groups of participants were recruited. The ART group consisted of HIV-infected adults with a suppressed viral load for ≥1 year on ART, the viremic group consisted of HIV-infected individuals not taking ART with detectable VL, and the healthy control (HC) group was comprised of age- and sex-matched HIV-uninfected controls. HIV-infected individuals were recruited from the Chronic Viral Illness Service of the McGill University Health Centre. The exclusion criteria included acute respiratory or viral illness within two weeks of NO testing, chronic pulmonary disease, or current use of nasal/inhaled steroids or nasal decongestants. The participants were in the clinic 2 hours prior to NO testing, during which time there was no exercise, nor any food or drink consumption.

Individuals completed the Sinonasal Outcome Test-20 (SNOT-20) questionnaire, a widely used and validated quality-of-life instrument that assesses the clinical impact of chronic rhinosinusitis [4]. The clinical and laboratory parameters on HIV infection within three months of enrollment were collected from medical records. Nasal NO testing was performed by standardized methods using a CLD88 chemiluminescence analyzer (EcoPhysics, AG, Duernten, Switzerland) [5–7]. While seated with a plastic catheter and soft nasal sponge placed securely into one nostril, patients blew into a cardboard resistor in order to close their velum and prevent dilution of nasal gas (containing much higher NO levels) with gas from lower airways. Expiratory maneuvers lasted at least 10 seconds, producing an initial washout phase followed by a NO concentration plateau phase, signifying steady state NO sampling from the nasal cavity. The same procedure was done for the contralateral nostril. For each nostril, the mean of 2 separate plateau measurements were calculated, followed by the mean for both nostrils together to provide the final result [5, 6]. Nasal NO values are expressed in nanolitres/minute (nL/min), which reflects the product of the nasal NO concentration in parts per billion and the flow rate in the sampling catheter of 0.33 L/min. All NO measurements were performed with ambient NO levels <50 ppb. Descriptive and inferential analyses were performed using SPSS 23.0 and graphs were created using GraphPad Prism 7.0. Comparisons were tested at 5% level of significance using independent sample *t*-test, *χ*^2^ test, Fisher's exact test, and one-way analysis of variance (ANOVA) with LSD post hoc test where appropriate. Correlations were measured using Spearman's rank correlation coefficient. Multivariate linear regression analysis was conducted to adjust for the effect of age, gender, body mass index (BMI), eosinophil count, and time of NO measurement on nasal NO levels.

## 3. Results

A total of fifty-eight (58) individuals participated, including 41 (71%) individuals with undetectable VL and 5 (9%) viremic individuals off ART. In addition, 12 (21%) healthy, HIV-uninfected volunteers also participated in the study. The majority (76%) of participants were tested between 11 am and 5 pm, when nasal NO values are in steady state and unaffected by normal circadian changes in nasal NO [8].

The mean age of participants was 47 ± 10 years old and 62% were males. The distribution of age and sex was statistically similar across the three groups (*p* > 0.05). None of the participants had previously undergone nasal operations or sinus procedures, and none had any known history of nasal polyps. Participant characteristics are outlined in Table 1. A significantly larger proportion of individuals (42%) with HIV infection on ART were smokers compared to HIV-infected individuals off ART (20%), while none of the healthy controls were smokers (*p* = 0.012, Fisher's exact test). Furthermore, none of the healthy controls nor viremic HIV-infected individuals off ART reported marijuana use, whereby significant proportions of HIV-infected participants on ART reported marijuana use (37%) (*p* = 0.008, Fisher's exact test).

**Figure 1:**
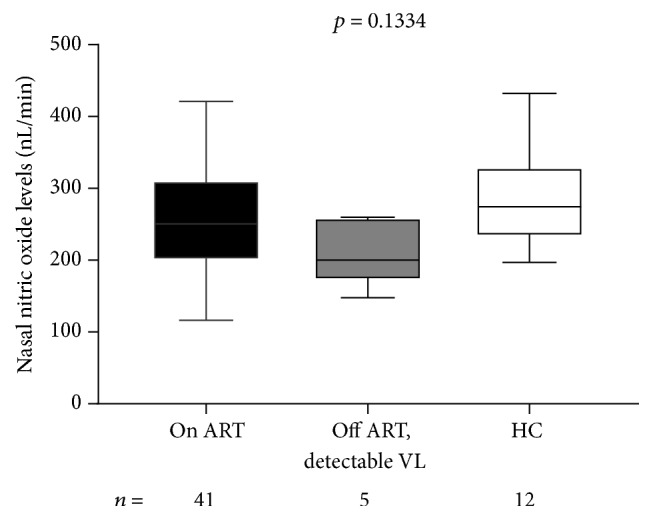
*Nasal NO levels across the 3 groups of participants*. Mean nasal NO levels in HIV-infected individuals with suppressed viral load on ART (black bar), HIV-infected individuals with detectable viral loads (VLs) off antiretroviral therapy (ART) (grey bar), and healthy controls (HC, white bar). The differences of mean nasal NO levels among groups did not reach statistical significance.

Figure 1 depicts mean nasal NO levels between the 3 groups. Healthy controls had the highest mean nasal NO levels (289 ± 68 nL/min), followed by HIV-infected ART-treated patients (253 ± 77 nL/min) and HIV-infected viremic individuals not on ART (213 ± 48 nL/min). However, the overall differences of mean nasal NO levels among the groups did not reach statistical significance (ANOVA, *p* = 0.133) despite a trend towards decreased nasal NO levels in viremic individuals off ART. In post hoc analysis, decreased nasal NO levels in viremic individuals off ART compared to healthy controls were nearly significant (*p* = 0.057). However, multivariate analysis confirmed no difference of nasal NO levels in untreated versus ART-treated HIV-infected persons adjusting for clinical factors such as age, sex, time of NO measurement, BMI, and absolute eosinophil count. The multivariate analysis could not include healthy controls owing to missing information in several variables such as body mass index and absolute eosinophil count.

We did not find any associations between nasal NO levels and age, current CD4 counts, CD4/CD8 ratio, CD4 nadir, duration of HIV seropositivity, smoking status, ART regimen, or SNOT-20 score (all *p* > 0.05). Similar correlation analyses of NO levels by group categories also showed insignificant results. Furthermore, the difference in mean nasal NO levels between participants tested between 11 am and 5 pm versus 5.01 pm and 10.59 pm did not significantly differ (*t*-test, *p* = 0.543).

As shown in Table 2, mean SNOT-20 scores significantly differed among groups (ANOVA, *p* = 0.013). HIV-infected individuals on ART had significantly higher (indicating worse sinonasal disease symptoms) mean SNOT-20 scores than healthy controls (19 ± 16/100 versus 4 ± 4/100, LSD post hoc test *p* = 0.004). There were significantly higher proportions of individuals with ≥15 points on the SNOT-20, indicative of moderate-to-severe symptoms, in HIV-infected individuals on ART (54%) and HIV-infected individuals off ART (40%) versus healthy controls (0%, *p* = 0.001; Fisher's exact test). Furthermore, a greater proportion of individuals on ART with suppressed VL reported experiencing at least 2 symptoms together ≥7 times per year, which is more often than individuals with detectable VL off ART versus healthy controls (*p* < 0.001; Fisher's exact test, Table 2).

**Figure 2:**
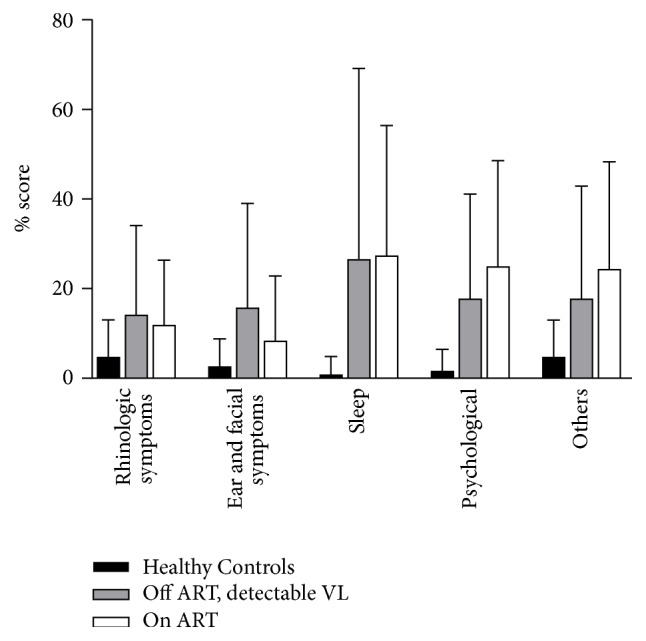
*SNOT-20 questionnaire scores across the 3 groups of participants*. Participants with HIV infection both on and off ART had higher total scores on the questionnaire (*p* = 0.013). This difference was observed in all domains, although higher scores among HIV groups were driven by sleep and psychological symptoms more than rhinologic or ear and facial symptoms.

Figure 2 depicts SNOT-20 scores for the 3 groups broken down by domain and expressed as a percentage for the possible total score in that domain (i.e., a score of 5 out of possible 25 points was expressed as 20%). For all domains, HIV+ participants had higher scores than the healthy control group, although the higher SNOT-20 scores observed in the HIV groups were driven more by sleep and psychological symptoms than by rhinosinusitis or ear and facial symptoms. HIV+ participants both on and off ART also had high scores in the “Others” category which asks about cough and whether the individual wakes up tired.

## 4. Discussion

For the first time in the modern ART era, we examined nasal NO levels in HIV-infected individuals on ART with suppressed VLs and HIV-infected individuals off ART with detectable VLs. We did not find statistically significant differences in nasal NO levels between these groups and age/sex-matched healthy controls. However, participants with HIV infection have increased clinical symptoms from chronic rhinosinusitis as measured by the SNOT-20 questionnaire.

In the cross-sectional study by Palm and colleagues from 2001, nasal NO levels were measured in 31 HIV-infected individuals and 26 controls [3]. Of the persons with HIV, 28 were on ART and 7 previously had an AIDS-defining illness. The average CD4 count was 320 ± 40 cells/mm^3^ (CD4%  20 ± 1.8) and average viral load was 26,300 ± 14,560 copies/ml. Nasal NO was 21% lower in HIV patients than controls (152 ± 11.4 in HIV patients versus 193 ± 16.7 nL/min in controls, *p* = 0.004). In our study, HIV-infected individuals on ART and off ART had much higher mean NO values (253 ± 77 nL/min and 231 ± 48, resp.) than found by Palm et al. This variation may result from our nasal NO measurement technique, which was slightly different from the technique used by Palm and colleagues, as even modest changes in nasal NO measurement protocols can result in significantly different final values [9]. However, our higher nasal NO values are more likely from baseline immunologic differences and data analysis schema in the studied populations. First, Palm and colleagues analyzed nasal NO values from individuals with detectable and undetectable VL as one single group, which may have masked the differences we observed between these two populations. Additionally, the mean CD4 count in our study population was higher at 546 and 496 cells/mm^3^ in persons on and off ART, respectively, and 50% of participants in both of our groups had CD4 ≥500 cells/mm^3^. Furthermore, our HIV study populations had significantly higher CD4/CD8 ratios (mean ± std 0.817 ± 0.51 in individuals on ART and 0.52 ± 0.280 in individuals off ART) compared with the participants in Palm et al. (0.41 ± 0.05), which is indicative of greater immune reconstitution in our population. These differences between CD4/CD8 ratios of our HIV participants both on and off ART versus those in the Palm et al. study were statistically significant (*p* < 0.001 and *p* = 0.040, resp.). The higher CD4 and CD4/CD8 ratios in our study may reflect the current practice to initiate ART sooner in the course of HIV than was done in the past years. The average viral load in our HIV+ participants off ART was 27,752 ± 24,477 copies/ml, which is similar to that of the patients in the study by Palm et al. Overall, our findings suggest that HIV suppression mediated through ART is associated with improvements in the immune system including nasal NO production, although differences observed between our groups on and off ART were not statistically significant. We did not observe any significant correlations between various patient characteristics and nasal NO levels in HIV-infected individuals. Our lack of ability to detect correlations between nasal NO levels and various clinical parameters, such as CD4 count, may be due to our small sample size or due to the fact that there truly is no correlation between these parameters and nasal NO levels in HIV-infected adults.

Like other studies performed in HIV-infected populations, we noted a high proportion of individuals who reported tobacco and/or marijuana smoking [10–12], but we did not find any significant correlation between smoking and nasal NO levels, nor did we find a correlation between marijuana smoking and nasal NO levels in our study. In HIV-uninfected populations, tobacco smoking has been associated with increased nasal NO levels [13, 14]. Zhou et al. found that active smokers with high urine cotinine levels had significantly increased nasal NO levels compared to both nonsmokers and nonsmokers exposed to second-hand smoke [14]. In an observational cohort study of individuals undergoing functional endoscopic sinus surgery for management of persistent chronic rhinosinusitis, both smokers and nonsmokers had similar preoperative SNOT-20 scores [15]. However, prior smoking history was associated with smaller improvements in postsurgical quality-of-life SNOT-20 scores than in nonsmokers [15]. In another study examining the effect of smoking on outcomes after endoscopic sinus surgery, average SNOT-16 scores were significantly higher (indicating worse disease symptoms) in smokers versus nonsmokers [16]. In patients undergoing endoscopic sinus surgery for chronic sinusitis, additional studies have also shown that tobacco smoking negatively impacts patient-reported outcomes and quality-of-life measures in smokers versus nonsmokers, although not all of these studies employed the SNOT questionnaire [17–19]. In our study, we observed similar SNOT-20 scores in never, past, and current smokers.

To our knowledge the association between marijuana smoking and nasal NO levels has never been previously examined. In a study examining illicit drug use and various health conditions, marijuana smoking was associated with bronchitis but not sinusitis [20]. Whether marijuana smoking could have artificially increased nasal NO levels in our HIV-infected population on ART, who had significantly higher rates of marijuana smoking than the other two groups, is a matter of speculation. Studying the associations between smoked marijuana and nasal NO levels may also be confounded by the fact that individuals who smoke marijuana may also smoke tobacco and possibly other substances.

A major limitation of our study is our small sample size of 5 individuals off ART. Given the awareness of the benefits of treating HIV as early as possible in the course of infection [21], very few individuals off ART presented to our clinic. Furthermore, when patients who were lost to follow-up did return to clinic after a period of absence with ART noncompliance, they often had major psychosocial stressors prohibiting participation in a research protocol. Many other patients who presented to the clinic after a long period of absence were quickly reinitiated on ART during that same visit. We also did not examine participants for nasal polyps or deviated septum. However, a deviated septum should not matter for this study as we averaged the values for the 2 nostrils together to obtain a final nasal NO level. Furthermore, for healthy controls we were missing information such as body mass index, eosinophil count, CD4 count, and CD4/CD8 ratio. Therefore, the multivariate analysis could not include healthy controls owing to the missing information.

Our reliance on a retrospective chart review for clinical and laboratory data is another limitation. For many patients diagnosed in the 1990s and early 2000s, it was not clear if the lowest CD4 counts found in the charts were truly the nadir CD4 counts. Finally, ideally we would have measured inflammatory markers and associations with nasal NO levels; however, resource constraints precluded this additional testing. Despite these limitations, this study provides insight into a relatively unexplored area of immunology in people living with HIV both on and off effective ART in the modern ART era.

## 5. Conclusion

Respiratory tract immunity is important for preserving health and quality of life in HIV patients. Though previously thought to have decreased nasal NO values, HIV-infected patients on ART, as well as a small population of HIV-infected viremic individuals off ART, have nasal NO levels similar to healthy controls. Despite this, HIV-infected individuals have increased clinical symptoms from chronic rhinosinusitis as measured by the SNOT-20 questionnaire. Further investigation into immune disturbances of the respiratory tract in individuals on and off ART is needed to elucidate reasons for increased rhinosinusitis in this population and targets amenable to intervention.

## Figures and Tables

**Table 1:** Characteristics of study participants.

*Characteristics or outcome*	HIV+ with suppressed VL on ART (*n* = 41)	HIV+ with detectable VL off ART (*n* = 5)	Healthy controls (*n* = 12)	*p*
Age, years [mean (std)]^#^	48 (10)	45 (9)	43 (12)	0.327

Male sex [*n* (%)]^$^	28 (68%)	3 (60%)	5 (42%)	0.297

Duration of HIV infection, years [mean (std)]^~^	16 (9)	10 (8)	- -	0.209

Antiretroviral regimen [number (%)]^1^; NRTIs and				
PI	11 (27%)	NA	NA	NA
NNRTI	10 (24%)			
Integrase inhibitor	27 (66%)			
Cell-entry inhibitor	1 (2%)			

Duration of viral load suppression, years [mean (std)]	6 (3)	NA	NA	NA
Duration of detectable viral load years [mean (std)]	NA	3 (3)	NA	NA

CD4 count (cells/*μ*l)				
All patients [mean (std)]^~^	543 (301)	496 (195)	- -	0.737
Patients with CD4 ≤350 [*n* (%)]^$^	16 (39%)	1 (20%)		0.674
Patients with CD4 351–499 [*n* (%)]	5 (12%)	1 (20%)		
Patients with CD4 ≥500 [*n* (%)]	20 (49%)	3 (60%)		
CD4% [mean (std)]^~^	31 (12)	25 (6%)		0.256
CD8 count (cells/*μ*l), [mean (std)]^~^	809 (452)	1041 (331)		0.275
CD4/CD8 ratio [mean (std)]^~^	0.817 (0.509)	052 (0.278)		0.210

Nadir CD4 count (cells/*μ*l) [mean (std)]	203 (147)	280 (166)	- -	0.329

Viral load, VL; standard deviation, std; nucleoside reverse transcriptase inhibitor, NRTI; protease inhibitor, PI; nonnucleoside reverse transcriptase inhibitor, NNRTI; integrase inhibitor, II; not applicable, NA; ^1^percentages surpass 100% as some individuals were on more than 1 drug class in addition to a NRTI backbone; statistical tests used for comparison: ^#^ANOVA; ^$^Fisher's exact test; ^~^independent sample *t*-test.

**Table 2:** Nasal nitric oxide levels and responses to items on the impact of Sinonasal Outcomes Test-20 (SNOT-20) questionnaire.

*Characteristics or outcome *	HIV+ with suppressed VL on ART (*n* = 41)	HIV+ with detectable VL off ART (*n* = 5)	Healthy Controls (*n* = 12)	*p*
Nasal nitric oxide levels (nL/min) [mean (std)]^#^	253 (77)	213 (48)	289 (68)	0.133
Nasal nitric oxide levels (nl/min) [median (IQR)]	251 (200, 311)	200 (172, 259)	274 (233, 330)	
Range in nitric oxide levels (nL/min)	116–421	147–264	197–432	
Participants with levels ≤300 nL/min, [*n*(%)]^$^	29 (71%)	5 (100%)	7 (58%)	0.291

Total points on the SNOT-20 questionnaire (100 possible) [mean (std)]^#^	19 (16)	18 (26)	4 (4)	**0.013**
Participants with total points 0–14^$^ (no problem to mild problem) [*n* (%)]	19 (46%)	3 (60%)	12 (100%)	**0.001**
Participants with ≥15 points (moderate to severe) [*n* (%)]	22 (54%)	2 (40%)	0 (0%)	**0.001**
Range in points	0–59	0–60	0–13	
Number of times per year a person experiences ≥2 symptoms in the SNOT-20 together [*n*(%)]^$^				
Never	9 (24%)	2 (40%)	7 (58%)	**<0.001**
1-2 times	4 (11%)	1 (20%)	1 (8%)	
3-4 times	1 (2%)	0 (%)	4 (33%)	
≥7	24 (63.2%)	2 (40%)	0 (0%)	
Question left blank	3 (7%)	0 (0%)	0 (0%)	

Statistical tests used for comparison: ^#^ANOVA and ^$^Fisher's exact test.
